# Supplementary material for: The impact of HIV-1 subtypes on virologic and immunologic treatment outcomes at the Lagos University Teaching Hospital: A longitudinal evaluation
Source: PLoS One. 2020 Aug 25;15(8):e0238027. doi: 10.1371/journal.pone.0238027 (PMC7447033; doi:10.1371/journal.pone.0238027)
Supplement: S1 Fig — (DOCX) [file pone.0238027.s001.docx]

| 42 had no sequence data  29 had no clinical data  240 antiretroviral-naïve people enrolled in LUTH site  VL  I69 included in study  CD4  83  3month  92  86  86  6 months  81  83  105  67  64  9 months  60  12 months  115  109  101  24 months  68  99  98  36months  71  83  110  48months  59  60  147  60 months  46  22 |
| --- |

S1 Fig: Study profile showing number of participants included in analysis at each time line.

Number of participants included at each timeline. Number of participants with missing CD4+ cell count results. No of participants with missing VL results
