# Supplementary material for: The impact of HIV-1 subtypes on virologic and immunologic treatment outcomes at the Lagos University Teaching Hospital: A longitudinal evaluation
Source: PLoS One. 2020 Aug 25;15(8):e0238027. doi: 10.1371/journal.pone.0238027 (PMC7447033; doi:10.1371/journal.pone.0238027)
Supplement: Attachment — Submitted filename: Response to reviewers.doc [file pone.0238027.s002.doc]

29^th^ May 2020

To the Editor

PLOS ONE

Dear Sir,

LETTER OF REBUTTAL:

The Impact of HIV-1 Subtypes on Virologic and Immunologic Treatment Outcomes at the Lagos University Teaching Hospital: A longitudinal evaluation (PONE-D-19-29623)

Thank you inviting us to submit a revised version of the manuscript that addresses the points raised during the review process. We are honored that your journal feels that our manuscript has merit.

We have done this and attached it below in a table format.

Thanks you.

Yours Sincerely

Ann Abiola Ogbenna

|  | Comment | Correction | Page |
| --- | --- | --- | --- |
| Academic editor | | | |
| 1. | It is unclear if the 169 participants included represent all patients at this hospital that met the enrollment criteria or a subset of those individuals. How representative are these 169 individuals of the HIV-positive population at this hospital? In Nigeria? | 240 adult patients aged >18 years were recruited over a period of one year. Of these, 198 participants had a baseline sequence data out of which only 169 had clinical follow up data to interrogate the impact of subtypes on treatment outcomes.  A detailed flow chart has been included as supplementary data | Page 5  Lines 6, 108,  Page 6  116-127 |
| 2. | A more robust discussion of the potential limitations is warranted. | In line with the Editor’s review, we have revised and elaborated on the limitations of the study. | Page 14  Lines 249-253 |
| 3. | The authors should who the sequence data as a phylogenetic tree. | This present study is a secondary data analysis. We have now described the primary study in the methodology section. Reference of the primary study was also included. | Page 6  Line 119-125 |
| 4. | How do the authors know that a sufficient sample size was included to robustly evaluate if subtypes differ in disease progression? | This was a programme study and baseline sequence data was conducted for the participants. Our study is a secondary data analysis of all available complete data. Though the primary study for the sequence data was a prospective cohort study and appropriate sample size was calculated based on: virologic outcomes after 24 months on ART, a 20% loss to follow up and a 25% mortality rate after 24month. The fact that sample size was not calculated initially to include a 5-year follow up remains a limitation of the study. This has been alluded to in page 14, line 252-259. | Page 14  Line 252-259 |
| 5. | Please provide additional details regarding participant consent. In the ethics statement in the Methods and online submission information, please ensure that you have specified whether consent was written or verbal/oral. If consent was verbal/oral, please specify: 1) whether the ethics committee approved the verbal/oral consent procedure, 2) why written consent could not be obtained, and 3) how verbal/oral consent was recorded. If your study included minors, please state whether you obtained consent from parents or guardians in these cases. | A written consent was obtained for all participants |  |
| Reviewer 1 | | | |
| 1 | The PLOS Data policy requires authors to make all data underlying the findings described in their manuscript fully available without restriction, with rare exception (please refer to the Data Availability Statement in the manuscript PDF file). The data should be provided as part of the manuscript or its supporting information, or deposited to a public repository. For example, in addition to summary statistics, the data points behind means, medians and variance measures should be available. If there are restrictions on publicly sharing data—e.g. participant privacy or use of data from a third party—those must be specified. | See above |  |
| 2 | Methods: The time points need to be mentioned here and this will help with the reporting of the time line in the results section. There is also no mention of how many samples were shipped to Johannesburg for testing by each time point. Page 5 | Data for serial CD4 count and viral loads were extracted from electronic data base at baseline, 3/12, 6/12, 9/12, 12 months, 24 months, 36months, 48 months and 60month. This has been reflected in the manuscript    Samples were shipped in 3 batches. This also has been reflected in the methodology | Page 6,  Lines 116-119 |
|  | Results |  |  |
| 3 | The results were very confusing to follow and understand, but this should be sorted once the time points are clearly stated in the methods section. I suggest that flow diagram showing sample availability at each of the tested time points be included and reasons for samples missing and those with missing data (Page 7). For example, in Table 3 the researchers list possible reasons why samples are missing but even in those that were still under care, the numbers reported don’t add up to the totals in the table (Page | Time points have been stated in methodology and  Flow chart added as a supplementary data.  Data on those still in care was obtained from drug pick up data.  However, it was not possible to determine why these patients had missing laboratory results as such records are not kept in the clinic. |  |
| 4 | Separate reporting of results for viral load and CD4 counts it will be clearer when reporting in the results section. Also report them by time points so that it is clear to see the rise in CD4 or viral load by time point tested. (Page 9 and 10) | Table 2 has been separated for VL and CD+ count and reported by timelines  A favorable immunologic response was defined as CD4+ cell count rise of ≥100 copies/µL from baseline CD4+ cell count within 12 months, hence table 2a shows this.  Viral suppression was defined as HIV RNA levels ≤ 1000 copies/mL. This is reflected in table 2b and the title has been changed to reflect the content | Page 8 and 9 |
| 5 | There also needs to be consistency in the number of visits, in Figure 1 there are 9 time points for CD4 count while Table 2 and 3 show that you had 2 visits (Page 8 and 9). For viral load there are 4 visits as shown in Table 2 and Table 3 (Page 8 and Page 9). | Table 2 shows as title reflects outcome at first 12 months hence the fewer number of visits, while figure 1 shows outcome over the whole period of study hence 9 time points  Table 3 as title reflects shows outcome at end point only i.e.  month 60 |  |
|  | discussion |  |  |
| 6 | There is inconsistencies in the CD4 counts of those infected with CRF 06-cpx and CRF 18-cpx subtypes (Figure 1). The authors did not show why this was happening and this happened for just these two subtype. The low numbers that they mentioned in the results section does not explain such (Page 9). | In line with the Reviewer’s comment, we have revised and elaborated on probable reasons. | Page 9  Line 176-179 |
| Reviewer 2 | | | |
| 1 | Reference #14 is not in the text but is stated in the reference list. | Stated in P4 line 84 now | Page 4  Line 84 |
| 2 | In this manuscript, Ogbenna and colleagues longitudinally followed up 169 study participants infected with different subtypes who were started on antiretroviral therapy in Nigeria. The authors report that the individuals did not differ in baseline CD4+ T cell counts or viral loads according to HIV-1 subtype, and there were no differences in immunological and virological outcomes following initiation of antiretroviral therapy. Overall, this is a sound study although I found the report generally poorly written and sometimes confusing. | We have gone through the manuscript and included time lines as stated above for clarity. |  |
| 3 | Do the authors have CD4% and CD4:CD8 ratios for participants in the cohort? Others have reported that CD4% and CD4:CD8 ratios do not normalize even after long-term antiretroviral therapy in other cohorts so it would be interesting to see whether that holds true for this cohort and if there are differences in these measures by HIV-1 subtype. | No, we do not have CD % or CD4: CD8 ratio. |  |
| 4 | In table 1, only two antiretroviral drugs are shown for participants in different categories. Were participants in this cohort on two-drug regimens instead of three? Why is that the case? | No they were on 3 drugs. however, they were classified based on NRTI backbone. | Page 6  Line 156 |
| 5 | In all the tables, there is repetition in data presentation- why for example show the numbers of both males and females? Why not just show one category (with percentage) since for these binary variables it is obvious what the numbers and percentages will be for the other category? The same applies for measures such as CD4+ T cell count increases above 100 cells/µl, viral load suppression below 1,000 copies/ml, etc. | There is no repetition of data. Table titles have been edited to represent content. | Table 2a: Page 8  Table 2b: Page 9 |
| 6 | The authors should show the confidence intervals for the data presented in figures 1-3. Also is this the same data in tables 1-4 and only presented differently? It would be preferable that there is no data repetition unless there is a specific separate point that the authors want to highlight by presenting the same data in different formats. | Not the same data.  Figure 1 shows the median CD4+ cell count over the whole period of study. Table 2a show proportion with a favourable immunologic response at 12 months after initiation of therapy, while table 2b shows proportion with viral suppression at variable times within the first 12 months.  Table 3 shows outcome at end of study period which is 60^th^ month  CI were reported as prose page 10, 176-182 |  |
| 7 | The figure legends for figures 1 to 3 are mixed up with the manuscript results narrative. The figure legends should be separated from the results narrative to make it easier to follow. | This has been rectified  Journal requires that figure legend be placed at place where it first mentioned in article, though it is uploaded separately. | Page 8, line 169 |
| 8 | Although the REGA tool was used for subtyping, a more robust phylogenetic analysis with reference sequences would have been useful, particularly considering the extreme diversity of viral strains in Nigeria | As explained above, the data sequence data was a secondary data. This has been stated and referenced in the methodology. |  |
| 9 | The conclusion that a higher proportion of CRF-06-cpx and CRF-18-cpx remained in care at 60 months seems tenuous and implies that there are differences between subtypes in terms of response to treatment. Unless the authors can show that this is a solid finding backed by systematic data rather than a chance finding, it should be removed from the abstract. | This has been removed from abstract | Page 2  Lines 44&45 |
