# Supplementary material for: The impact of HIV-1 subtypes on virologic and immunologic treatment outcomes at the Lagos University Teaching Hospital: A longitudinal evaluation
Source: PLoS One. 2020 Aug 25;15(8):e0238027. doi: 10.1371/journal.pone.0238027 (PMC7447033; doi:10.1371/journal.pone.0238027)
Supplement: Attachment — Submitted filename: Response to reviewers_PONE-D-19-29623R1.doc [file pone.0238027.s003.doc]

06^th^ August 2020

Jason Blackard

Academic Editor

PLOS ONE

Dear Sir,

LETTER OF REBUTTAL:

The Impact of HIV-1 Subtypes on Virologic and Immunologic Treatment Outcomes at the Lagos University Teaching Hospital: A longitudinal evaluation (PONE-D-19-29623R1)

Once again we thank you and the reviewer for the time, care and effort put into reviewing our manuscript. We are grateful for the generous comments of the reviewers.

We gave gone over all the points raised and addressed them. We thank the reviewer for pointing out the need to indicate the third ARV used by participants. This has been addressed in result section; page 8, line 160-161.

We have also reflected the confidence intervals in the graph as suggested. GraphPad prism software was used to plot them. This has been reflected in the methodology; page 7, lines 145 & 146.

We thank the reviewer for spotting the discrepancy in the supplementary figure. This was a typo error; 86 had viral loads and 83 (not 88) did not have viral loads. This correction has been effected in the supplementary figure.

An annotated version of the manuscript and a clean unmarked version has been uploaded into the journal website.

All figures have been uploaded into PACE. Figures 2 and 3 met PLOS ONE requirement, whereas the TIF file for figure one was converted to a valid TIF file for PLOS ONE.

I hope that we have done justice to these minor edits raised by reviewer 2. We thank you in advance for your continuous support and consideration for our manuscript.

Thanks you.

Yours Sincerely

Ann Abiola Ogbenna

Senior Lecturer

College of Medicine, University of Lagos

Nigeria.
